# Association of operative approach with adverse events following hepatic resections

**DOI:** 10.1016/j.sopen.2025.09.004

**Published:** 2025-09-08

**Authors:** Arjun Chaturvedi, Esteban Aguayo, Oh. Jin Kwon, Kevin Tabibian, Barzin Badiee, Saad Mallick, Daniel Tabibian, Peyman Benharash

**Affiliations:** a Center for Advanced Surgical and Interventional Technology (CASIT), David Geffen School of Medicine, University of California, Los Angeles (UCLA), Los Angeles, CA, USA; b Harbor-UCLA Surgery Center, Torrance, CA, USA; c Department of Surgery, David Geffen School of Medicine, UCLA, Los Angeles, CA, USA

**Keywords:** Hepatectomy, Operative approach, Major resection, Outcomes

## Abstract

**Background:**

Despite growing use of minimally invasive surgery (MIS) for hepatocellular carcinoma (HCC), contemporary national data contrasting MIS with open hepatectomy are sparse.

**Methods:**

Adults (≥18 y) undergoing hepatectomy for HCC in the American College of Surgeons National Surgical Quality Improvement Program (2015–2022) were studied. MIS (laparoscopic or robotic) resections were compared with open operations. Entropy balancing harmonized covariates, and multivariable logistic or linear models produced adjusted odds ratios (AOR) for major adverse events (MAE; composite of mortality and serious complications), liver-specific complications, and 30-day readmission.

**Results:**

Among 5832 hepatectomies, 27.0 % were MIS, rising from 18.8 % in 2015 to 36.1 % in 2022 (*p* < 0.001). After adjustment, MIS was associated with markedly lower odds of MAE (AOR 0.36, 95 % CI 0.29–0.46), postoperative liver failure (AOR 0.34, 0.20–0.58), bile leak (AOR 0.49, 0.28–0.89), need for invasive intervention (AOR 0.29, 0.18–0.47), and 30-day readmission (AOR 0.61, 0.44–0.86). In the subset undergoing major resections, MIS retained protective associations for MAE (AOR 0.26, 0.15–0.46) and readmission (AOR 0.28, 0.11–0.80).

**Conclusions:**

In a large, contemporary U.S. cohort, MIS hepatectomy was independently associated with fewer perioperative complications, liver-specific adverse events, and readmissions compared with open surgery—even for major resections. These findings support continued expansion of minimally invasive hepatectomy and targeted training to extend its benefits to appropriately selected patients with resectable HCC.

## Highlights

• Minimally invasive hepatectomy use doubled between 2015 and 2022.
• MIS was associated with lower odds of major adverse events following hepatectomy.
• Even among major resections, MIS remained independently associated with improved perioperative outcomes.
• MIS hepatectomy offers a safe and effective alternative to open surgery, supporting its broader adoption.

## Introduction

Hepatocellular carcinoma (HCC) is the sixth most common malignancy globally and the third leading cause of cancer-related deaths nationally [1]. Surgical resection offers the only widely available curative option for patients with resectable disease, yet hepatectomy is technically demanding and carries substantial risk of adverse events, including hemorrhage, bile leak, and liver failure [2,3]. Over the past two decades, the emergence of minimally invasive surgery (MIS), including laparoscopic and robotic approaches, has sought to mitigate these hazards by enhancing visualization, reducing surgical trauma, and hastening recovery [4]. Early series and meta-analyses have suggested that MIS can achieve comparable oncologic margins while decreasing perioperative blood loss, postoperative pain, and length of stay [5,6]. Consequently, the adoption of MIS hepatectomy has risen steadily, particularly at high-volume hepatobiliary centers [7].

Despite these encouraging trends, the comparative safety of MIS and open hepatectomy remains incompletely defined, especially for complex or major resections. Prior investigations have largely been limited to single-institution cohorts which lack contemporary risk adjustment and focus on a narrow subset of outcomes [8,9]. Moreover, previous studies have excluded higher-risk populations such as older adults, patients with cirrhosis, or those requiring neoadjuvant therapy, groups increasingly encountered in modern practice [9]. As a result, the generalizability of existing evidence to routine clinical care is uncertain, and the magnitude of any perioperative advantage conferred by MIS on nationally representative population has yet to be clarified.

The present study uses the American College of Surgeons National Surgical Quality Improvement Program (ACS NSQIP) to evaluate temporal trends in MIS utilization and to examine the association between operative approach and short-term clinical outcomes following hepatectomy for HCC. We hypothesized MIS to be independently associated with lower odds of MAE, liver-specific complications, and 30-day readmissions compared with open resection.

## Methods

**Fig. 1:**
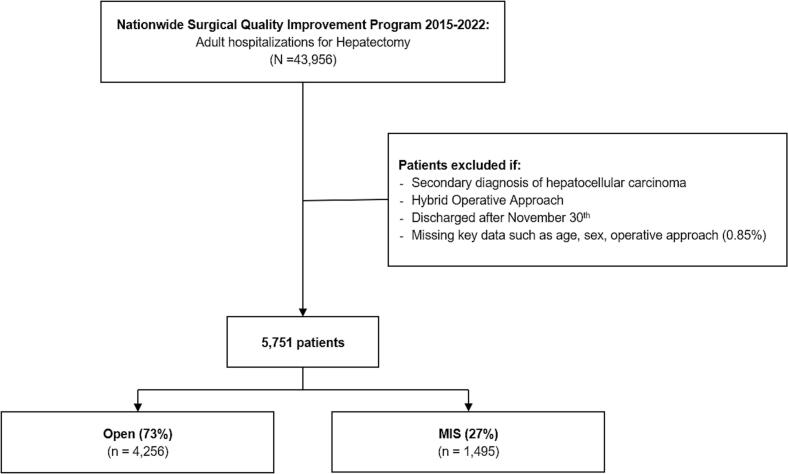
Consort Diagram of study cohort and survey-weighted sample size. MIS, Minimally Invasive Techniques.

All adult (≥18 years) hospitalizations for patients undergoing hepatectomy were identified in the 2015 to 2022 American College of Surgeons' National Surgical Quality Improvement Program (NSQIP) Targeted Hepatectomy Participant User Files using relevant Current Procedural Terminology (CPT) codes [10]. ACS NSQIP collects over 150 demographic, operative, and medical characteristics as well as 30-day postoperative outcomes from over 850 participating hospitals. Each contributing hospital conducts regular data collection and quality control audits with the help of a designated NSQIP-trained reviewer. Only patients with a primary diagnosis of hepatocellular carcinoma were considered for analysis. Records with a hybrid surgical approach, those who were discharged after November 30th of each year, or were missing key data were not considered (Fig. 1).

Patient characteristics were defined using ACS NSQIP-provided data elements and included age, sex, race, functional status (independent, partially dependent, totally dependent), and disease staging was performed following Tumor, Node, and Metastasis (TNM) staging [11]. Hepatectomy specific comorbidities were likewise tabulated from the NSQIP data elements accounted for preoperative neoadjuvant therapy as well as intraoperative findings of cirrhosis and hepatitis. The Model for End-Stage Liver Disease Score (MELD Score), a previously validated metric for quantifying liver disease severity, was further employed to estimate operative risk. Minimally invasive surgery (MIS) was defined as hepatectomy performed using either a laparoscopic or robotic approach. Hepatic resections were classified as either standard or major resections, with major resections being defined as the removal of 3 or more segments of the liver or an entire lobe during surgery.

Major adverse events were defined as a composite of in-hospital mortality as well as common surgical complications that included cardiac, neurological, infectious, gastrointestinal, thromboembolic, and respiratory sequelae [12]. Liver specific complications were composed of postoperative liver failure, bile leak, and invasive intervention. Briefly, invasive intervention was defined as any unplanned procedure, excluding reoperations, required to manage perioperative complications.

The primary outcome of interest was major adverse events. Secondary endpoints included liver specific complications and 30-day all cause readmissions.

Continuous variables were summarized as medians with interquartile range (IQR) or means with standard deviations (SD), while categorical variables were reported as proportions (%). Medians and proportions were compared across groups using the Mann-Whitney U and Chi-Squared tests, respectively. Cuzick's nonparametric test was applied to determine the significance of temporal trends (nptrend) [13]. Entropy balancing is a reweighting methodology that was used to account for intergroup differences by achieving a covariate balance between groups [14]. Unlike propensity score matching, this methodology identifies a set of sample weights that meet predetermined balance criteria while preserving the entire study population for analysis [15]. Multivariable logistic and linear regression models were fit to evaluate independent associations between MIS status and study end points. Covariates were selected using elastic net regularization, which minimizes collinearity through a penalized least squares methodology [16]. Models were evaluated using receiver operator curves and calibrations plots as needed. Regression outputs were reported as either adjusted odds ratio (AOR) or beta coefficients (β) for logistic and linear models with 95 % confidence intervals (CI) respectively. An α of 0.05 was set for statistical significance. All statistical analysis was performed on Stata 16.1 software (StataCorp, LLC, College Station, TX). This study was deemed exempt from full review by the Institutional Review Board at University California, Los Angeles.

## Results

**Fig. 2:**
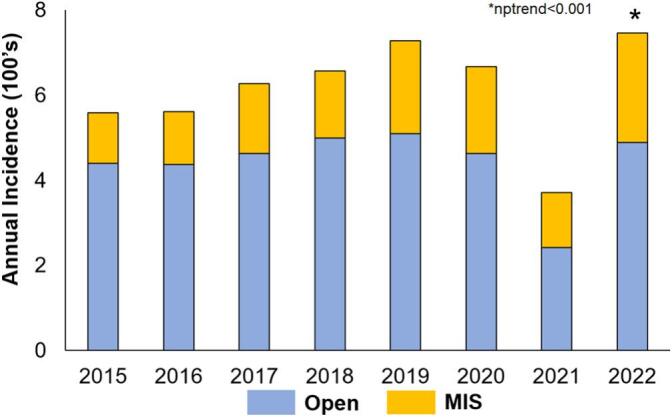
Annual trends in Minimally Invasive Surgery Utilization. The rate Minimally Invasive Surgery usage increased significantly over the study period, nptrend <0.001.

Of an estimated 5832 hepatic resections included in our analysis, 27.0 % (1495) were performed using MIS. The total number of MIS procedures more than doubled during the study period (119 procedures in 2015 vs 257 in 2022; nptrend < 0.001; Fig. 2). Compared to others, MIS patients were on average older (67 IQR: [61, 73] vs 66 years [59, 72, *P* < 0.001), more likely to be female (31.8 vs 26.5 %, P < 0.001), and less often of the white race (47.4 vs 52.3 %, *P* < 0.001; Table 1). Patients undergoing MIS were less frequently classified as ASA class IV (71.0 vs 74.3 %, *P* < 0.001), but presented with higher rates of hepatitis (55.4 vs 48.2 %, P < 0.001) and intraoperative findings of cirrhosis (49.7 vs 31.2 %, *P* < 0.001). Moreover, MIS patients demonstrated a slightly lower MELD score (7.5 IQR: [6.5, 8.5] vs 7.8 IQR [6.7, 8.7]; *P* = 0.03) compared to their open counterparts. MIS patients were also less likely to receive preoperative neoadjuvant therapy (5.6 vs 12.5 %, *P* < 0.001) and less frequently underwent a major resection (12.1 vs 40.3 %, *P* < 0.001). Among neoadjuvant therapy recipients, MIS patients were less likely to receive locoregional interatrial infusions (1.6 vs 2.6 %; *P* < 0.001), portal vein embolization (0.6 vs 3.9 %; *P* < 0.001), and preoperative systemic chemotherapy (1.9 vs 2.5 %; P < 0.001). Finally, the MIS cohort was more likely to be diagnosed as T1 (61.4 vs 43.8 %; *P* < 0.001), N0 (6.5 vs 8.7 %; *P* < 0.001), and M0 (99.4 vs 98.6 %; P < 0.001) cancer stages compared to their open counterparts.

**Table 1:** Demographic and clinical characteristics.

	Open (*n* = 4256)	MIS^a^ (*n* = 1495)	*P*-value
Patient characteristics			
Age (years, median [IQR])	66 [59, 72]	67 [61, 73]	<0.001
Female (%)	26.5	31.8	<0.001
MELD Score (units, median [IQR])	7.5 [6.5, 8.5]	7.8 [6.7, 8.7]	0.03
Race (%)			<0.001
White	52.6	47.4	
Black	10.5	9.6	
Asian or Pacific Islander	17.9	23.3	
Other/Unknown	19.0	19.7	
ASA Classification (%)			0.03
No Disturbance	0.5	0.6	
Mild Disturbance	16.0	19.1	
Severe Disturbance	74.3	71.0	
Life Threatening	9.2	9.5	
Function (%)			<0.001
Independent	99.1	98.0	
Partially Dependent	0.9	1.9	
Totally Dependent	0.02	0.13	
Comorbidities (%)			
Chronic Obstructive Pulmonary Disease	5.6	7.1	0.09
Coronary Heart Failure	0.8	0.9	0.79
Diabetes	29.1	30.2	0.66
Dyspnea	8.1	9.8	0.66
Hypertension	60.4	61.6	0.44
Smoker	21.8	23.3	0.24
Steroid Use	3.1	2.3	0.11
Weight Loss	3.8	2.3	0.02
Hepatitis	48.2	55.4	<0.001

Perioperative characteristics			
Liver Texture (%)			<0.001
Normal	21.2	15.3	
Fibrosis	4.5	3.2	
Fatty	9.5	8.0	
Congested	1.1	0.9	
Cirrhotic	31.2	49.7	
Not Documented	32.5	22.9	
Neoadjuvant Therapy (%)			<0.001
None	91.0	95.9	
Locoregional interatrial infusions	2.6	1.6	
Portal vein embolization	3.9	0.6	
Preoperative systemic chemotherapy	2.5	1.9	
Pringle Maneuver (%)	32.2	20.4	<0.001
Hepatic Ablation (%)	9.4	8.7	0.44
Major Resection (%)	13.0	6.8	<0.001

Cancer staging			
Clinical T stage (%)			<0.001
T0	0.6	0.4	
T1	43.8	61.4	
T2	35.8	30.1	
T3	13.1	5.1	
T4	6.6	2.8	
Clinical M Stage (%)			<0.001
M0	98.6	99.4	
M1	1.4	0.6	
Clinical N Stage (%)			<0.001
N0	91.1	93.5	
N1	8.7	6.4	
N2	0.2	0.1	

Reported as proportions unless otherwise noted. Statistical significance was set at α = 0.05.

a MIS, Minimally Invasive Surgery; IQR, interquartile range.

Upon bivariate comparison, MIS patients less often experienced a major adverse event (14.2 vs 31.7 %, *P* < 0.001) and were readmitted less frequently (6.7 vs 10.4 %, P < 0.001). Additionally, the MIS cohort exhibited lower rates of postoperative liver failure (1.9 vs 7.2 %, P < 0.001), invasive intervention (3.3 vs 9.3 %, P < 0.001), and bile leak (2.4 vs 6.4 %, *P* < 0.001; Table 2) compared to their open counterparts.

**Table 2:** Unadjusted outcomes of patients undergoing Hepatic Resection, stratified by MIS status.

	Open (n = 4256)	MIS (n = 1495)	*P*-value
Clinical outcomes (%)			
Major Adverse Events	37.0	16.7	<0.001
Invasive Intervention	9.3	3.2	<0.001
Bile Leak	6.4	2.4	<0.001
Liver Failure	7.1	2.3	<0.001

Resource utilization (%)			
Nonelective 30-day readmission	10.4	6.7	<0.001

Outcomes reported as proportions. Statistical significance was set at α = 0.05.

**Fig. 3:**
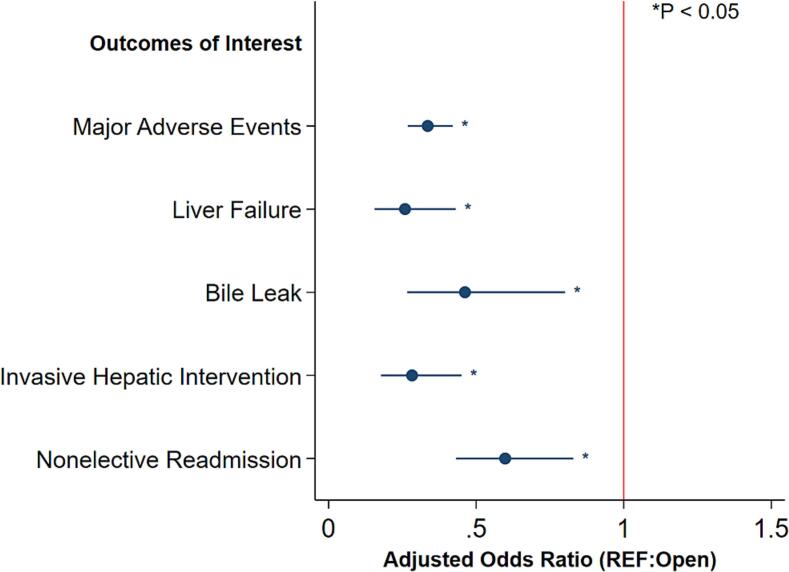
Association of Minimally Invasive Technique (MIS) status with selected outcomes of interest, with Open status as reference.

After entropy balancing, adequate covariate balance was achieved. Following risk adjustment, MIS status was associated with decreased odds of major adverse events (AOR: 0.36 95 % CI [0.29, 0.46], *P* < 0.001) as well as 30 day all cause readmissions (AOR: 0.61 95 % CI [0.44, 0.86], *P* < 0.001, Fig. 3). MIS was also associated with a reduced likelihood of postoperative liver failure (AOR: 0.34 95 % CI [0.20, 0.58], P < 0.001), invasive intervention (AOR: 0.29 95 % CI [0.18, 0.47], *P* < 0.001), and bile leak (AOR: 0.49 95 % CI [0.28, 0.89], P < 0.001; Table 3).

**Table 3:** Adjusted outcomes of patients undergoing hepatectomy, stratified by MIS status.

	AOR/β Coefficient (with 95 % CI)	*P*-value
Clinical outcomes (AOR)		
Major Adverse Events	0.36 [0.29, 0.46]	<0.001
Liver Failure	0.34 [0.20, 0.58]	<0.001
Invasive Intervention	0.29 [0.18, 0.47]	<0.001
Bile Leak	0.49 [0.28, 0.89]	0.02

Resource utilization (AOR)		
Nonelective 30-day readmission	0.61 [0.44, 0.86]	0.005

Outcomes reported as Adjusted Odds Ratio (AOR) or β Coefficient, with 95 % confidence intervals (CI).

Reference: Open

Model Covariates included were age, female sex, race, elective status, operative time, neoadjuvant therapy, hepatitis, intraoperative findings of cirrhosis, pringle maneuver utilization, ASA class, preoperative creatinine, preoperative bilirubin, congestive heart failure, chronic obstructive pulmonary disease, smoking status, dialysis status, diabetes mellitus, and ascites.

**Fig. 4:**
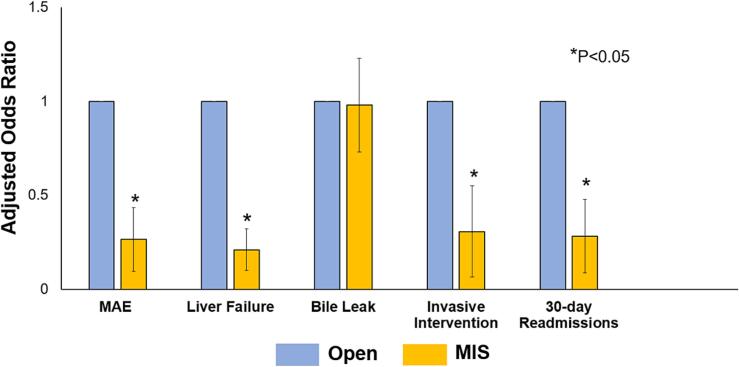
Association of Minimally Invasive Technique (MIS) status with selected outcomes of interest for patients undergoing major hepatic resections, with Open status as reference.

An additional subgroup analysis was performed solely on patients undergoing major hepatic resections. Following risk adjustment, MIS was associated with decreased odds of MAE (AOR: 0.27 95 % CI [0.15, 0.46], P < 0.001) and 30 day all cause readmissions (AOR: 0.28 95 % CI [0.11, 0.80], *P* = 0.02, Fig. 4). MIS status was also associated with reduced risk of postoperative liver failure (AOR: 0.21 95 % CI [0.08, 0.59], *P* = 0.003) and need for invasive intervention (AOR: 0.31 95 % CI [0.11, 0.86], *P* = 0.03), but similar odds of bile leak (AOR: 1.06 95 % CI [0.35, 3.21], *P* = 0.92; Table 4).

**Table 4:** Adjusted outcomes of patients undergoing Major Hepatic Resection, stratified by MIS status.

	AOR/β Coefficient (with 95 % CI)	*P*-value
Clinical outcomes (AOR)		
Major Adverse Events	0.26 [0.15, 0.46]	<0.001
Liver Failure	0.21 [0.08, 0.59]	0.003
Invasive Procedure Needed	0.31 [0.11, 0.86]	0.03
Bile Leak	1.06 [0.35, 3.21]	0.92

Resource utilization (AOR)		
Nonelective 30-day readmission	0.28 [0.11, 0.80]	0.02

Outcomes reported as Adjusted Odds Ratio (AOR) or β Coefficient, with 95 % confidence intervals (CI).

Reference: Open

Model Covariates included were age, female sex, race, elective status, operative time, neoadjuvant therapy, hepatitis, intraoperative findings of cirrhosis, pringle maneuver utilization, ASA class, preoperative creatinine, preoperative bilirubin, congestive heart failure, chronic obstructive pulmonary disease, smoking status, dialysis status, diabetes mellitus, and ascites.

## Discussion

In the present work, we characterized the association of operative approach with select acute clinical outcomes following hepatectomy and made several significant observations. Hepatectomy utilization increased during the study period, with MIS usage nearly doubling. Moreover, MIS status was associated with decreased odds of MAE, liver-specific complications, and a reduction in the 30-day readmission rate compared to their open counterparts. In a sub-group analysis of only patients undergoing a major hepatic resection, MIS was associated with decreased odds of MAE and 30 day-all cause readmissions. Several of these findings warrant further discussion.

Congruent with prior literature, the total hepatectomy case volume increased significantly between 2015 and 2022. The rise in hepatic resection utilization can be explained by several factors including improvements in procedural safety as well as a concomitant upsurge in the national prevalence of hepatocellular carcinoma [17,18]. Better understanding of liver anatomy through expanded residency education, coupled with improvements in preoperative care, have contributed to enhanced national hepatectomy use [19,20]. Additionally, the centralization of cases to high-volume tertiary centers has been attributed to further improving safety, as these institutions offer greater operator experience along with expanded access to laparoscopic and robotic surgical platforms [21]. Reflecting this institutional shift, MIS usage nearly doubled during the study period. This trend can best be attributed to a confluence of factors including expanded residency training and decreased instrumentation costs [22]. A 2021 cross sectional study found a 19 % increase in the number of minimally invasive surgery fellowships between 2008 and 2019, improving operator experience nationally for hepatectomy [23]. Furthermore, Pawlik and colleagues noted MIS instrumentation costs have consistently decreased allowing for greater institutional access for laparoscopic and robotic surgical techniques [10]. Together, these developments reflect a national shift towards laparoscopic and MIS surgical platforms for hepatectomy.

Our study further noted minimally invasive techniques to be associated with decreased odds of experiencing postoperative complications as well as a reduction in 30-day all cause readmissions. These findings are in line with previous literature, with Pawlik and colleagues reporting patients undergoing MIS for hepatectomy to have a lower incidence of morbidity [10]. Likewise, a meta-analysis of 12 studies focused on outcome differences between open and laparoscopic hepatectomy, observed a significant decrease in postoperative morbidity and a shortened hospital stay for patients undergoing MIS techniques [24]. The reported benefits of minimally invasive hepatectomy can be imputed to several underlying rationale including improved surgical precision as well as the use of hemihepatic occlusion of vascular inflow [25,26]. Meticulous dissection, often facilitated by minimally invasive techniques, has been shown to improve procedural safety by minimizing inadvertent tissue trauma and vascular injury [27]. Moreover, Nanashima and colleagues reported that the increased usage of advanced hemostatic devices in laparoscopic platforms has reduced the incidence of postoperative bile leak and hemorrhage [28]. Collectively, these findings demonstrate that minimally invasive surgery is a safe and effective method for hepatectomy.

Furthermore, MIS utilization was associated with a reduction in major adverse events as well as a decrease in liver-specific complications for patients undergoing major hepatic resections. In this context, MIS procedures and specifically robotic-based interventions may provide some advantages in facilitating increased precision during surgical dissection and micro suturing during complex biliary reconstruction [[29], [30], [31], [32], [33]]. Prior work has shown that the expanded imaging capabilities associated with minimally invasive surgery provides a magnified view of the operative field, allowing for enhanced visualization of key anatomical features particularly within the liver hilum, where the dense anatomy demands meticulous handling [29]. Additionally, the micro suturing capabilities provided by minimally invasive techniques are especially valuable in enabling the closure of small-caliber ducts in total biliary reconstruction [31]. Notwithstanding these potential benefits, MIS utilization remained rather limited in major reconstructions and less often employed inflow occlusion techniques intraoperatively [33]. Together, these findings support institutional adoption patterns that more commonly utilize MIS platforms, even in the case of large resections, for less technically demanding repairs and reconstructions rather than a uniform application of MIS techniques across all levels of surgical complexity [34].

This study has several important limitations, including those inherent to its retrospective design. Of note, the ACS NSQIP is derived from patient data collected at select, high volume, teaching hospitals. Therefore, our results are not generalizable to all centers in the country, where surgeon-specific differences in training, operative experience, and outcomes may exist. Outcomes in NSQIP are only recorded 30-days postoperatively, preventing further analysis beyond this period. Additionally, we were unable to ascertain certain granular clinical information, including the presence of vascular reconstruction and the location of resection. Consequently, the relative complexity of cases between the MIS and open cohort could not be fully ascertained. Nonetheless, the present study utilized robust statistical methodology to mitigate the impact of the present limitations.

The utilization of MIS hepatectomy has risen markedly over the study period and is associated with decreased odds of MAE and 30-day all cause readmissions. Minimally invasive techniques offer enhanced outcomes, supporting its continued adoption, even in cases of major resections. Expanding surgeon proficiency in laparoscopic and robotic techniques, alongside efforts to increase access to minimally invasive platforms, may further improve perioperative outcomes for patients with resectable HCC. However, prospective, long-term studies are warranted to better characterize the durability of these benefits and to inform optimal surgical management across diverse clinical settings.

## Funding/financial support

No direct or indirect financial support by extramural sources was received for this research.

## CRediT authorship contribution statement

**Arjun Chaturvedi:** Writing – original draft, Visualization, Methodology, Investigation, Conceptualization. **Esteban Aguayo:** Writing – review & editing, Visualization, Methodology, Investigation, Conceptualization. **Oh. Jin Kwon:** Writing – review & editing, Visualization, Methodology, Formal analysis, Conceptualization. **Kevin Tabibian:** Writing – original draft, Visualization. **Barzin Badiee:** Writing – review & editing, Data curation, Conceptualization. **Saad Mallick:** Writing – review & editing, Methodology, Investigation, Conceptualization. **Daniel Tabibian:** Methodology, Investigation. **Peyman Benharash:** Writing – review & editing, Supervision, Software, Project administration, Methodology, Investigation, Conceptualization.

## Ethics statement

This work complies with the standards put forth by Elsevier's Publishing Ethics Policy.

## Declaration of competing interest

All authors report no conflicts of interest or disclosures.
